# Additive Free Crosslinking of Poly-3-hydroxybutyrate via Electron Beam Irradiation at Elevated Temperatures

**DOI:** 10.3390/polym15204072

**Published:** 2023-10-12

**Authors:** David Krieg, Michael Thomas Müller, Regine Boldt, Mirko Rennert, Markus Stommel

**Affiliations:** 1 Institute for Circular Economy of Bio:Polymers at Hof University (ibp), 95028 Hof, Germany; mirko.rennert@hof-university.de; 2 Leibniz-Institut für Polymerforschung Dresden e.V., 01069 Dresden, Germany; mueller-michael@ipfdd.de (M.T.M.); boldt@ipfdd.de (R.B.);; 3 Institute of Material Science, Technical University Dresden, 01069 Dresden, Germany

**Keywords:** poly-3-hydroxybutyrate, irradiation, e-beam, crosslinking, chain branching, chain scissioning, elevated temperatures, molten state, melt, gel point, gel dose, G values, biopolyesters

## Abstract

When applying electron or gamma irradiation to poly-3-hydroxybutyrate (P3HB), main chain scissions are the dominant material reactions. Though propositions have been made that crosslinking in the amorphous phase of P3HB occurs under irradiation, a conclusive method to achieve controlled additive free irradiation crosslinking has not been shown and no mechanism has been derived to the best of our knowledge. By applying irradiation in a molten state at 195 °C and doses above 200 kGy, we were able to initiate crosslink reactions and achieved gel formation of up to 16%. The gel dose D_gel_ was determined to be 200 kGy and a range of the G values, the number of scissions and crosslinks for 100 eV energy deposition, is given. Rheology measurements, as well as size exclusion chromatography (SEC), showed indications for branching at doses from 100 to 250 kGy. Thermal analysis showed the development of a bimodal peak with a decrease in the peak melt temperature and an increase in peak width. In combination with an increase in the thermal degradation temperature for a dose of 200 kGy compared to 100 kGy, thermal analysis also showed phenomena attributed to branching and crosslinking.

## 1. Introduction

Poly-3-hydroxybutyrate (P3HB) is a bio-based and biodegradable polyester that can be derived from various carbon sources, including sewage waste, by more than 300 different bacteria [1,2,3,4]. Along with bacteria, different types of algae have been discovered, which are able to synthesize P3HB, opening the possibility for P3HB to be obtained from carbon dioxide and sunlight alone [5]. This makes P3HB a promising candidate for more sustainable polymer applications. However promising P3HB seems, when it comes to its sustainable production process, a lack of sufficient mechanical and thermal properties prevents a wider application range [6]. Compared to conventional plastics and even most other bioplastics, P3HB is more brittle [7]. P3HB’s elongation at break reaches from 1.4 to 8%, tensile strength from 20 to 40 MPa and its Young’s modulus lies between 0.28 and 4 GPa compared to other biopolyesters such as polylactic acid (PLA) and polycaprolactone (PCL), where values for elongation at break can reach 13% and 457%, tensile strength can reach 53 and 22 MPa and a Young’s modulus 2058 and 181 GPa, respectively, can be observed [7,8,9]. This is a result of poly-3-hydroxybutyrate’s high crystallinity, reaching up to 70% [10,11]. With a glass transition temperature around 0 °C, pushing to lower degrees of crystallinity through processing does not work, since cold crystallization increases the material’s crystallinity to its maximum within several days or weeks [12]. When it comes to processing P3HB, the proximity of the melting point (~175 °C) to its thermal degradation temperature (~195 °C) is problematic as well [13]. A processing step, e.g., compounding, can lead to a decrease in the molar mass by 25% or more, further decreasing the melting point and the thermal degradation temperature [13]. Hence, material modifications are necessary for P3HB to be applied to more polymer solutions.

**Figure 1:**
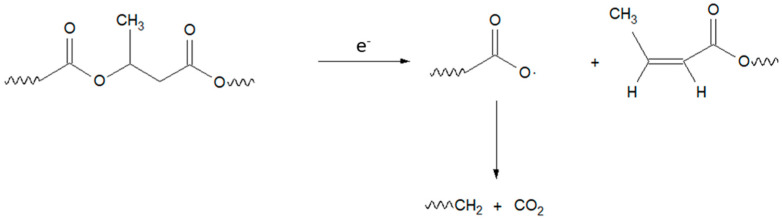
Schematic depiction of scissioning in P3HB under irradiation after [15].

For polymer modification, different options are at researchers’ disposal. For the enhancement of thermal and mechanical properties, additives can be compounded into the matrix material. Chemical modifications, such as copolymerization, are also possible. Additionally, polymers could be blended to combine the desired properties of different materials. Aside from the previously mentioned modification possibilities, ionizing irradiation such as electron beam (e-beam) or gamma irradiation can be utilized to induce changes in polymer materials [14]. Ionizing irradiation creates radicals in polymer materials. These radicals result from ionization, excitation, and electron capture of the radiation by the atoms in the organic molecules. These dislocated radicals can lead to branching, crosslinking, and chain scissioning of polymer chains. Whether crosslinking or chain scissioning is the dominant reaction under irradiation depends mostly on the mobility of the radicals and polymer chains, as well as the binding energies of atoms in the polymer backbone. For higher mobility, crosslinking becomes more dominant. For lower mobility, scissioning increases. Chain and radical mobility are influenced by the polymer chain structure, e.g., the presence of side groups or chains, the degree of crystallinity, and chain length. Weak points also influence whether a polymer crosslinks or scissions under irradiation. Weak points in the polymer backbone are created by side groups and chains. Side groups and chains weaken the chemical bonds in the main chain next to their attachment point. Therefore, they create a weak point for chain scissioning. The methyl side group in P3HB creates a weak point in the backbone. A schematic of the scissioning process for P3HB under irradiation is depicted in Figure 1 [15]. This, in combination with its high crystallinity and therefore limited chain mobility, hinders radical recombination and scissioning is reported to be the dominant reaction for the irradiation of P3HB in its high-crystalline, solid state [16]. Therefore, a drop in the molar mass and mechanical properties, as well as a glass transition and melting point temperature, can be seen [15,16].

It has been shown in the literature that the usage of additives and crosslink promoters or unsaturated monomers can help to overcome the difficulties regarding crosslink formation under irradiation [17,18]. It was also shown that the presence of plasticizers such as poly(ethylene glycol) (PEG) can result in crosslink formations in P3HB under irradiation [17]. Another way of promoting crosslink reactions is by lowering the crystallinity of P3HB. The first approaches to achieve this were made by irradiating P3HB at different times after processing before the maximal crystallinity was reached through cold crystallization [11]. It was argued that for irradiation with doses below 33 kGy shortly after processing, crosslinking occurs in the nearly amorphous material. A next logical step is the irradiation without any crystallinity of P3HB, e.g., in the molten state. Irradiation in the molten state ensures maximum polymer chain and polymer backbone radical mobility resulting from the absence of any crystalline structures. However, due to the aforementioned proximity of the melting point to the degradation temperature, this approach creates problems of its own.

**Figure 2:**
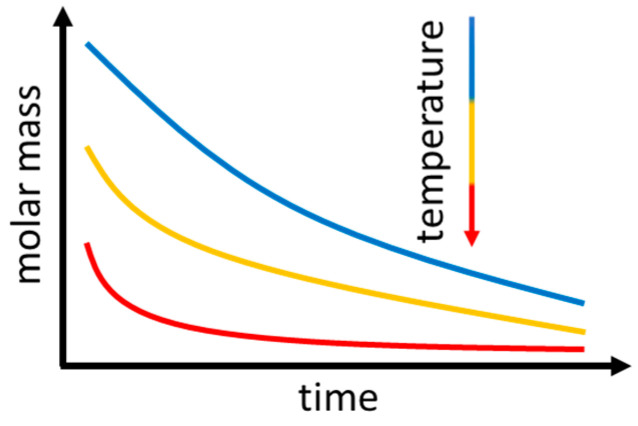
Schematic depiction of thermal degradation of P3HB depending on molar mass and temperature, according to [16].

**Figure 3:**
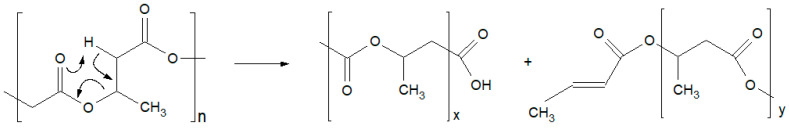
Mechanism of the cis-elimination in P3HB during thermal degradation according to [20].

Depending on the molar mass and temperature of the material, the severity of thermal degradation changes. A schematic depiction of the dependence of thermal degradation on the temperature and molar mass is shown in Figure 2 [19]. For lower molar masses, thermal degradation progresses faster. The same holds true for higher temperatures. During thermal degradation, P3HB undergoes a cis-elimination (see Figure 3) [20]. A cis-elimination describes a non-radical random chain scission reaction through the creation of a six-membered transition state [21,22].

This work investigates the irradiation of P3HB in a non-crystalline, molten state without crosslink agents. The melt was held at 195 °C and irradiated with 25 to 400 kGy via an electron beam under nitrogen. Through thermal analysis, gel content determination, as well as molecular mass, and rheological measurement we quantified the melting point decrease and the partial increase in degradation temperature, as well as crosslink formation and showed indications for branching. For all interpretations and conclusions, thermal degradation was considered. Through this methodology, a gap in science and the literature concerning the effect of the irradiation of P3HB at elevated temperatures is closed. This work places itself within the ongoing advances in science concerning the irradiation of biopolyesters above their glass transition temperature, in a molten state or while processing [23,24].

## 2. Materials and Methods

P3HB powder Enmant Y3000P was obtained from TianAn Biological Materials Co., Ltd. (Beilun Port, Ningbo City, China). According to the manufacturer, its specific density is 1.25 g/cm^3^, and it shows a melting temperature of 175 to 180 °C and a heat deflection temperature of 135 to 145 °C. The molar mass M_n_ of the virgin P3HB powder was determined by SEC to be around 1.2 × 10^5^ g/mol. The particle size of the powder d_90_ was 50 μm and the medium particle size diameter was 23 μm.

Irradiation was performed at the Leibniz-Institut für Polymerforschung Dresden e.V. (IPFDD) with an electron accelerator ELV-2 from Budker Institute of Nuclear Physics, Novosibirsk [25], Russia. Pre-dried P3HB powder was placed in circular molds with a diameter of 2.5 cm and a height of 0.2 cm, covered with a Kapton^®^ foil (thickness 125 µm), and placed in a special irradiation chamber [26]. After closing the chamber, the temperature was raised to 80 °C and kept under a vacuum for 5 min to remove moisture and oxygen. Subsequently, the irradiation chamber was flushed with nitrogen and the temperature was set to the target irradiation temperature of 195 °C and kept constant for 20 min during irradiation. Afterwards, the samples were cooled down to room temperature under nitrogen.

Before irradiation processing, samples were pre-dried in a vacuum oven at 40 °C at 0 mbar for 24 h. During the irradiation process, the electron beam parameters were kept constant at 1.5 MeV with an electron current of 4 mA. The irradiation doses ranged from 25 to 400 kGy applied in 25 kGy beam passage steps until the target dose was reached. From one mold, a sample mass of less than 2 g could be obtained. This was due to the low bulk density, below 0.3 g/cm^3^, of the sample material in powder form. The irradiated reference samples at 25 °C were produced the same way with no heat applied. To compensate the heat input during irradiation, a gradual control of the temperature during heating, depending on the dose, was performed.

Prior to analysis, all captured radicals along the polymer chain or in the crystalline phase must decay to ensure there are comparable initial conditions in the materials. Electron spin resonance (ESR) measurements of the sample irradiated at room temperature with 300 kGy showed that all captured radicals decayed after 22 h.

Differential scanning calorimetry (DSC) was performed with a Polyma 214 from Netzsch (NETZSCH-Gerätebau GmbH, Selb, Germany). Sample masses of 5.0 ± 0.3 mg were heated from −20 to 200 °C with a heating and cooling rate of 10 K/min. Each measurement consists of two heating cycles. To eliminate thermal history, the second cycle was used for analysis and discussion. To ensure thermal equilibrium while measuring, the samples were held isothermally for 3 min at −20 and 200 °C during the measurement cycles. Samples were placed in a closed aluminum crucible with a pierced lid. The DSC measurements were performed in a nitrogen atmosphere. The degree of crystallinity was calculated according to Equation (1),

(1) $$X_{c}=\frac{{∆H}_{m}-{∆H}_{cc}}{{∆H}_{m}^{0}}\times 100\%$$

with ${∆H}_{m}$ being the enthalpy of the melting peak, ${∆H}_{cc}$ the enthalpy of the cold crystallization peak, and ${∆H}_{m}^{0}$ the theoretical melting enthalpy of 100% crystalline P3HB. For ${∆H}_{m}^{0}$, a value of 146 J/g was obtained from the literature [27]. DSC measurements were performed several days after irradiation, to ensure the decay of all radicals.

For thermal gravimetric analysis (TGA), a TG 209 F3 Tarsus from Netsch (NETZSCH-Gerätebau GmbH, Selb, Germany) was used. Sample masses of 5.0 ± 0.2 mg were heated from 50 to 600 °C with a heating rate of 10 K/min. Samples were placed in an open aluminum oxide crucible. The TGA measurements were performed in a nitrogen atmosphere. Before measurement, the samples were placed in a desiccator for 4 days to ensure the absence of moisture in the material. The turning points were determined through the maximum of the first derivatives. Mass losses were referenced to the total masses at 150 °C.

The number average molecular mass (*M_n_*) and mass average molecular mass (*M_w_*) were determined via size exclusion chromatography (SEC). P3HB was dissolved in chloroform at 60 °C. After cooling the solution to 25 °C it was placed in a high-performance liquid chromatography (HPLC) Series 1100 system by Agilent Technologies Inc., USA. The SEC was equipped with a 1 PL MIXED-B-LS (300 × 7.5 mm) with a 10 μm PS gel column and coupled with a refractive index detector. As a reference, polystyrene with a range from 580 to 2.851 × 10^6^ g/mol was used. Results were obtained by averaging three measurements.

Rheology measurements were performed with a rheometer HR 20 from TA instruments (USA). Due to small sample sizes, an 8.0 mm parallel plate geometry was used, and the gap was set to 45 μm. The small sample size was due to the low bulk density of the sample material in powder form. The frequency range for the measurements was 63 to 1 rad/s with 0.1% strain. The temperature was kept at 165 °C during the measurement. Frequency sweep measurements were performed from high to low frequencies. A strain sweep was performed to ensure measurements were performed on the Newtonian plateau. All measurements were performed in air.

Gel content measurements were performed via Soxhlet extraction with chloroform (anhydrous, ≥99.8%) around its boiling point at 61 °C. Sample masses of 0.2 ± 0.02 g were placed in a type 390 filter paper (pore size 3–5µm). Contrary to the current state of the art, no stainless-steel mesh was used for sample containment during extraction, since containment of the P3HB powder could not have been ensured in a mesh. The extraction time was 6 h. After extraction, the samples were dried in a vacuum (0 mbar) at 60 °C for 8 h and then weighed. Gel content was calculated according to Equation (2)

(2) $$X_{gel}=\frac{m_{sample+paper}^{\prime }-m_{paper}}{m_{sampler+paper}-m_{paper}}\times 100\%$$

with $m_{sample+paper}$ being the combined mass of the sample and the filter paper before and $m_{sample+paper}^{\prime }$ after extraction. $m_{paper}$ labels the mass of the filter paper. From the soluble fraction obtained by the gel content measurements, the gel point and G values were determined via the Charlesby–Pinner Equation (3)

(3) $$s+\sqrt{s\;}=\frac{p_{0}}{q_{0}}+\frac{1}{q_{0}u_{1}}\frac{1}{D}$$

(4) $$s=1-\frac{X_{gel}}{100}$$

with s being the soluble fraction calculated according to Equation (4), $p_{0}$ and $q_{0}$ the density of scissions and crosslinks per unit dose, respectively, $u_{1}$ the number average degree of polymerization for the polymer of the most probable distribution of molecular weight, and D the irradiation dose in kGy [28,29]. The Charlesby–Pinner equation is only viable for polydispersities of two and lower. The polydispersities in this work ranged from 2.5 to 7, so applying the equation can only give an estimate of the true gel dose and G values. After simplifying Equation (3) in accordance with the literature [30], Equation (5) is obtained

(5) $$s+\sqrt{s\;}=\frac{G(S)}{2G(X)}+\frac{4.82\times 10^{6}}{G\left(X\right)\;Mn}\;\;\frac{1}{D}$$

with G(S) and G(X) being the number of scissions or crosslinks per 100 eV energy deposition in the material and Mn the mass of the polymer. The gel point is defined as an average of one crosslink unit per weight average molecule [30]. Therefore, Equation (5) simplifies to

(6) $$2=\frac{G(S)}{2G(X)}+\frac{4.82\times 10^{6}}{G\left(X\right)\;Mn}\;\;\frac{1}{D_{gel}}$$

where D_gel_ now describes where sudden changes in the molecular structure appear [31]. The Charlesby–Pinner plot was obtained, and solutions were obtained graphically. Uncertainties were calculated through error propagation with partial derivatives.

To determine the G values for the irradiation at room temperature, Equations (7) and (8) were used [32].

(7) $$\frac{1}{M_{nD}}=\frac{1}{M_{n0}}+1.037\times 10^{-7}\;\left(G\left(S\right)+G\left(X\right)\right)\;D\;\;$$

(8) $$\frac{1}{M_{wD}}=\frac{1}{M_{w0}}+1.037\times 10^{-7}\;\left(\frac{G\left(S\right)}{2}+2\;G\left(X\right)\right)\;D$$

*M_n_* is the number average molecular mass and *M_w_* the mass average molecular mass. The subscript 0 indicates the molar mass before irradiation, while the subscript D indicates the molar mass after a certain dose D was applied. The equations enable the calculation of the G values when no gel formation takes place [33]. For these equations to provide reliable values for G(S) and G(X), the polydispersity, the ratio of M_w_/M_n_, has to be equal to two. Since this is not the case for the material system used in this work, applying Equations (7) and (8) only gives an estimate of the true G values. The solutions to these equations were obtained graphically. Uncertainties for the G values were calculated through error propagation with partial derivatives.

## 3. Results and Discussion

### 3.1. Optical Inspection of Samples after Irradiation

Samples irradiated at room temperature showed no alteration in their optical appearance. The samples irradiated in the molten state as well as the reference sample that experienced the heating cycle for processing without irradiation showed signs of thermal degradation. The cooled down discs were very brittle, even to the point where they would partially break when taken out of the mold. This unfortunately made mechanical testing, e.g., with dynamic mechanical analysis (DMA), tensile testing, etc., impossible.

### 3.2. Thermal Analysis

**Figure 4:**
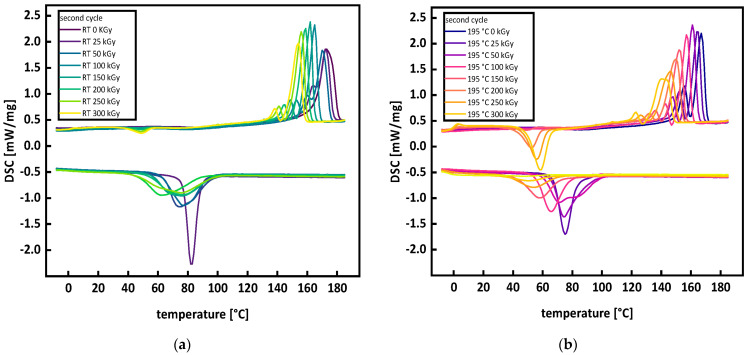
DSC curves of the second heating cycle (**a**) irradiation at room temperature and (**b**) irradiation in the molten state at 195 °C.

DSC measurements of P3HB show the development of a bimodal peak under irradiation at room temperature and in the molten state (see Figure 4a,b) because the molar mass decreases under irradiation due to random chain scission and leads to a bimodal distribution of crystallite sizes [16,17] (see 3.3 Molecular Mass and Branching). A second impact factor is the formation of unstable lamellar layers [17]. Da Silva et al. [34] also attributed the bimodal peak to different crystallite and lamella sizes as well as imperfections in the crystals, arguing that the peaks at higher temperatures are those of the more perfect crystals. Contrary to Oliveira et al. [16] and Parra et al. [17], irradiating with 5 to 300 kGy or 5 to 10 kGy, the bimodality of the peaks increased, and the total separation of the peaks was at 150 kGy for the irradiation at room temperature and at 100 kGy for the irradiation in the molten state. The P3HB used by Oliveira et al. [16] and Parra et al. [17] had a viscosity average mass of 360,000 g/mol or, respectively, an average molecular weight of 380,000 g/mol. The number average molar mass M_n_ of the P3HB used for this work was determined to be around 120,000 g/mol. Therefore, we argue the differences in the molar mass and the different susceptibilities of different molar masses to irradiation cause the discrepancy between this work and the works by Oliveira et al. [16] and Parra et al. [17]. The smaller initial molar mass of the P3HB used in this work may lead to a higher susceptibility towards scission, and therefore to different molar masses after irradiation, which results in different crystallization kinetics.

By comparing the DSC curves (see Figure 4a,b) of P3HB irradiated at room temperature and in the molten state for 0 kGy, it is evident that applying the heating cycle for irradiation processing in the molten state already leads to an increase in bimodality in the melting peak. This is due to the thermal degradation of P3HB during heating, leading to a more uniform molar mass distribution. For increasing doses, the peaks for the irradiation at room temperature become narrower, indicating a more uniform molar mass distribution. This is confirmed through SEC measurements (see Figure 9a): for the irradiation in the molten state, the main peaks widen (see Figure 4b). This is attributed to the molar mass distribution becoming multimodal (see Figure 9b) and is also a sign of crosslinking and branching as described by McKee et al. [35]. The assumption that the widening of the melting peak in the DSC curves is due to the occurrence of branching and crosslinking, is also supported by the data obtained from the SEC, gel content, and rheological measurements (see Figures 9b,12,13a).

**Figure A1:**
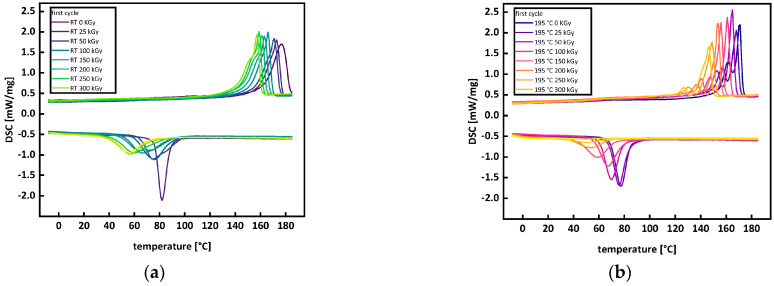
DSC curves of the second heating cycle (**a**) irradiation at room temperature and (**b**) irradiation in the molten state at 195 °C. For the first cycle.

All data obtained from DSC measurements for both heating cycles can be found in Appendix A, Figure A1 and Table A1.

**Figure 5:**
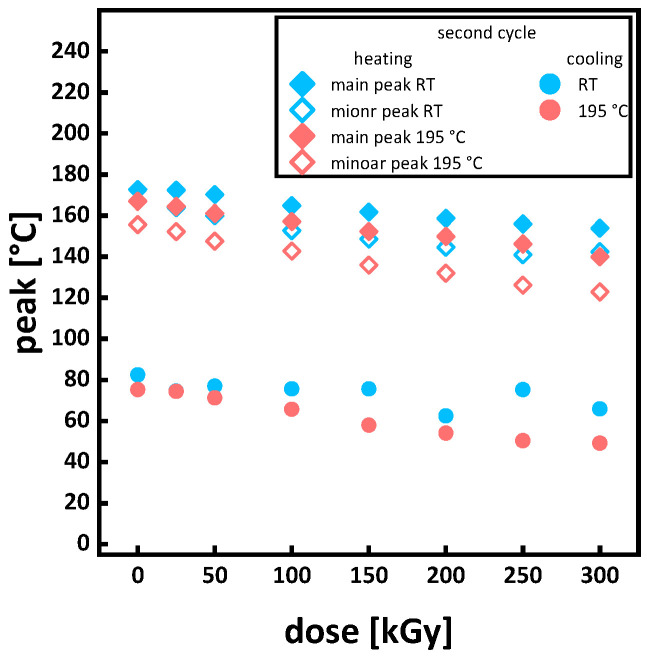
Melting points of the main and side peaks for heating and the main peaks for cooling from DSC measurements. Errors listed in the Table A1.

**Figure 6:**
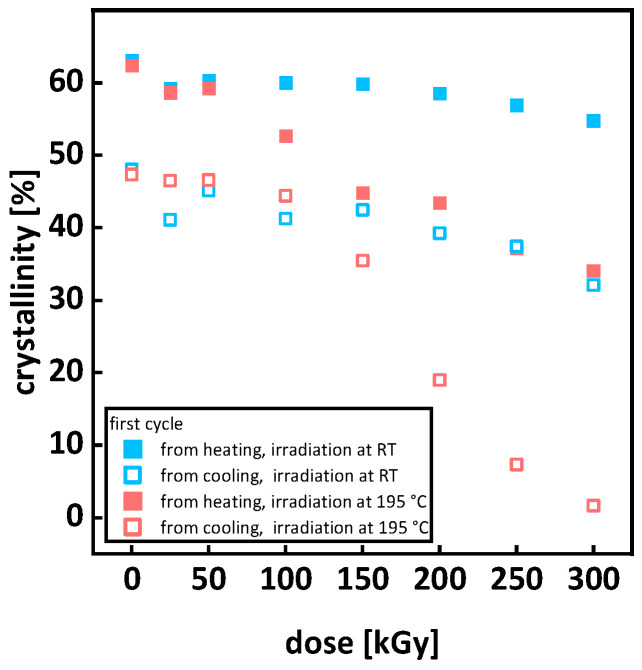
Crystallinity of P3HB under irradiation. The full boxes show crystallinity obtained from heating and the outlined boxes crystallinity obtained from cooling. Errors listed in the Table A1.

For a comprehensive overview, the changes in the melting points from heating and the crystallization points from cooling are extracted from the DSC curves and summarized in Figure 5. For increasing doses, the peaks of the DSC curves move to lower temperatures (see Figure 5). A linear decrease under irradiation is in agreement with the literature and is also attributed to the molar mass decrease under irradiation and its effect on the lamella structure and impurity of crystals [11,15,16,17,36]. The decrease in crystallinity is shown in Figure 6, where a higher drop in crystallinity is exhibited for irradiation in the molten state compared to room temperature. When comparing the decrease in crystallinity (see Figure 6) to the changes in molar mass (see Figures 10a and 11), it is evident that the decrease in crystallinity for the samples irradiated in the melt does not correlate with a decrease in the molar mass. The molar mass decrease by scission reduces chain entanglements, increasing chain mobility. Increased chain mobility leads to an acceleration of crystallization kinetics. Therefore, an increase in crystallinity was expected. Some of the literature reports that long chain branching (LCB) enhances the crystallinity [37]. Since our irradiated samples also contain amounts of long-branched chains, LCB may play a role in enhancing nucleation ability and leads, in our understanding, to a minor contribution to increased crystallinity. The lower crystallinity in the samples irradiated in the molten state is an indication for branching and crosslinking since structurally more complex molecule chains (e.g., star-like or tree-like branched) and networks hinder crystal formation. The formation of crosslinks and branching usually goes along with a widening of the melt peak and a decrease in the melt peak temperature [35]. The widening of the melt peaks, the decrease in crystallinity with no significant molar mass loss, as well as the decrease in the melting temperature point towards branching and crosslinking for the irradiation of P3HB in a molten state. This assumption is supported by the molar mass development (see Figure 9), rheological measurements (see Figure 12), and gel content development (see Figure 13a).

**Figure 7:**
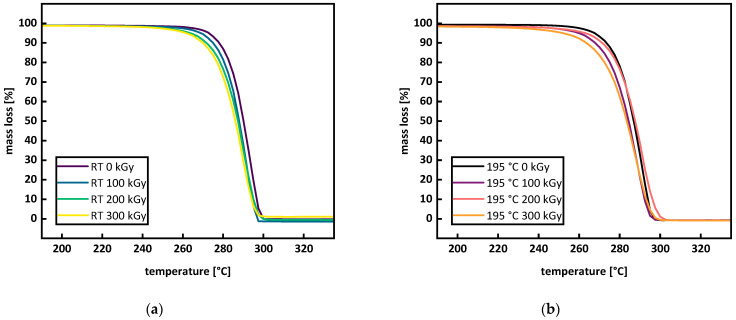
TGA curves of P3HB irradiated at room temperature (**a**) and in the molten state at 195 °C (**b**).

**Figure 8:**
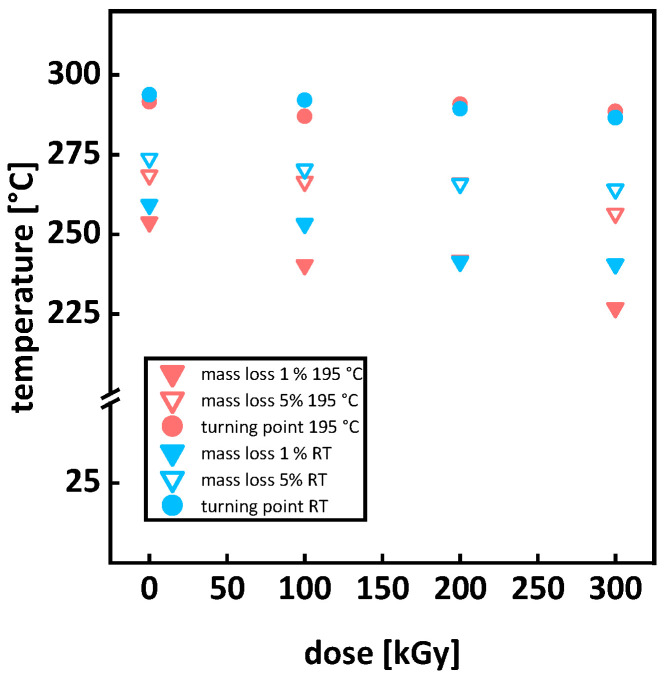
Temperature for different mass losses and turning points for P3HB irradiated at room temperature (blue) and in the molten state at 195 °C (red). At 200 kGy, data points overlap. Errors listed in the Table A2.

**Figure 9:**
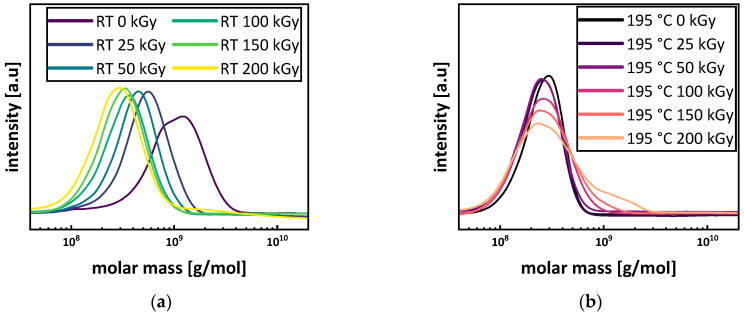
Molecular weight distribution of P3HB. (**a**) shows the changes in molar mass for the irradiation in the molten state at 195 °C and (**b**) for the irradiation at room temperature.

By examining the temperatures for predefined mass losses (e.g., 1 and 5%), changes in the starting point of thermal degradation can be quantified. This describes the course of thermal degradation in more detail and therefore consider the possible earlier degradation of smaller molar mass sections. Comparing the defined mass losses with the turning point of the degradation curve provides a picture of the whole thermal degradation process and not just the main degradation, which is mostly described by the inflection point of the first derivative. TGA measurements of P3HB irradiated at room temperature are shown in Figure 7a. For increasing doses, thermal degradation starts at lower temperatures. Figure 8 displays the temperatures for 1 and 5% mass loss, as well as the inflection point of the TGA curve. For irradiation at room temperature, a decrease in the temperature for mass loss with 1 and 5%, as well in the inflection point temperature, can be seen. A single-step thermal degradation process for the material system used in this work is in agreement with the literature [38], but for different types of P3HB, two-step thermal degradation processes can be found as well [39,40]. The mechanism of the thermal degradation of P3HB is displayed in Figure 3 and visualizes a cis-elimination, which is a non-radical random chain scission reaction through the creation of a six-membered transition state [21,22]. As depicted in Figure 9, the molar mass decreases under irradiation at room temperature and smaller molar masses lead to a faster and earlier thermal degradation (see Figure 2). This trend, however, is contrary to the trend described by Oliveira et al. [16]. They described an increased thermal stability from 293 °C (0 kGy) to 297 °C (300 kGy) through gamma irradiation. This opposite behavior could arise from the differences concerning the material states (molten or solid) during the irradiation or from the differences in dose rates using different radiation types, or might be due to different atmospheres during irradiation (nitrogen and air). For the irradiation in air, as performed by Oliveira et al. [16], the empirical constant for the determination of the viscosity average molecular weight M_v_ could be chaining. If this change was not taken into account for the calculations, an overestimation of the M_v_ values could occur. Overestimating M_v_ values will lead to higher G(S) values. Therefore, the potential for a molecular mass increase through branching and crosslinking is underestimated. Taking a closer look at the increase in thermal degradation temperature found by Oliveira et al. [16], this might have been the case, since an increase in the degradation temperature with increasing doses can be a sign of branching or crosslinking. There are also differences when it comes to radiation processing with electron or gamma irradiation. Gamma irradiation has a higher penetration capability than electron irradiation. Therefore, in the case of gamma irradiation, more time is needed to deposit the same energy into the polymer material when compared to electron beam irradiation. Electron beam irradiation increases the specimen’s temperature during irradiation more than gamma irradiation does, and therefore changes in the polymer chain and polymer backbone radical mobility can occur under electron irradiation. Electron irradiation also leads to charge deposition in the material. These differences between the radiation types can account for divergences in the material’s response. For polypropylene (PP), it was shown that gamma irradiation has a greater effect on the material than e-beam irradiation [41,42]. Hassan et al. [42] reported higher thermal degradation onset temperatures for gamma-irradiated PP at doses up to 60 kGy and a higher elastic modulus throughout gamma-irradiated test specimens, both being signs of a higher network formation in gamma-irradiated PP. Fintzou et al. [41] reported a stronger decline in the mechanical properties of PP during gamma irradiation compared to electron irradiation, also hinting towards higher crosslink formation during gamma irradiation. Unfortunately, neither work included gel content measurement for verification. It is argued that due to the usage of e-beam irradiation in this work, the increase described by Oliveira et al. [16] does not come into effect. During electron beam irradiation, the temperature in a specimen increases more than it does under gamma irradiation.

For the irradiation in the molten state, a shift to lower temperatures can be seen for 0 to 100 kGy. For a dose of 200 kGy, thermal degradation shifts to higher temperatures compared to 100 kGy, followed by a shift back to lower temperatures for 300 kGy (see Figure 7b and Figure 8). The turning point of the TGA decreased from 292 °C for 0 kGy to 287 °C for 100 kGy, then increased to 291 °C for 200 kGy, before decreasing to 289 °C for 300 kGy. For the temperature at 1% mass loss, a steady decrease from 254 to 227 °C with an increasing dose was determined. Temperatures for the 5% mass loss decreased from 269 to 266 °C for doses of 0 and 100 kGy. At 100 and 200 kGy, the temperature for the 5% mass loss did not change and decreased to 257 for 300 kGy. The shift to higher temperatures for thermal degradation at 200 kGy is attributed to the right balance of branching starting to occur at 100 kGy and the starting point of gel formation at 200 kGy (see Figures 9b and 13a). The network from gel formation in combination with branching leads to higher molar mass fractions that are more resistant to thermal degradation. Side chains, branching, and crosslinking possibly sterically hinder the chain mobility and movement; therefore, the cis-elimination may be reduced, which can lead to a higher thermal material resistance [43,44]. However, this effect does not change the overall thermal degradation process of P3HB [21]. The increase in thermal resistance correlates with the results obtained from rheological measurements. Therefore, the complex viscosity for doses of 150 to 250 kGy does not change, indicating a balance between branching and crosslinking (see Figure 12c). For 300 kGy, even though the maximal gel formation has been reached (see Figure 13a), the molecules not participating in the network are more sensitive to thermal degradation compared to those in the network. This is due to the branched or crosslinked molecules having a lower chain mobility, hindering them from undergoing a cis-elimination. Considering a network of only 16%, the overall thermal degradation is not dominantly influenced by the gel formation. The earlier thermal degradation of the lower molecular mass fractions that are not branched or incorporated in the network, maintained a balance with the later thermal degradation of the higher molar masses; therefore, no overall change in thermal degradation occurred. It is even possible that, when increasing doses over 250 kGy, the existing network is damaged. Crosslink density decreases and the distance between crosslink points increases. This explains the decrease in the thermal degradation temperature, even though gel content still exists. This proposition is backed up by rheological measurements (see Figure 12). The storage modulus and the complex viscosity decreased for doses of 100 kGy to 150 kGy and stayed unaffected for doses between 150 and 250 kGy before decreasing further for 300 kGy.

### 3.3. Molecular Mass and Branching

**Figure 10:**
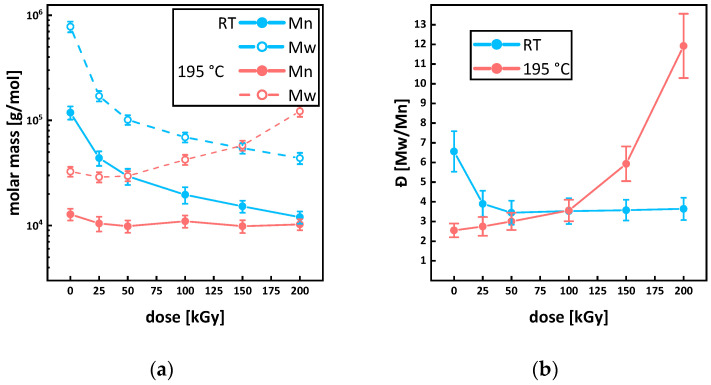
Number average molar mass M_n_ and mass average molar mass M_w_ (**a**) and polydispersity Ð for P3HB (**b**) irradiated in a molten state at 195 °C (red) and at room temperature (blue).

**Figure 11:**
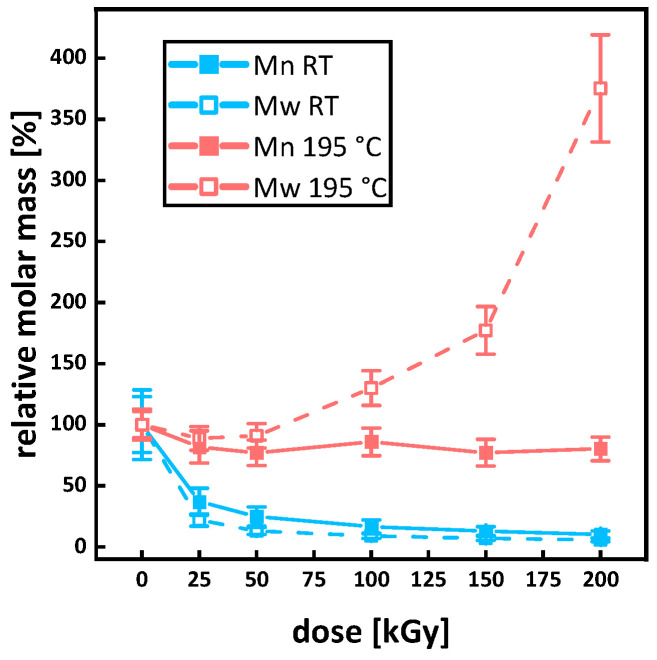
Relative molar mass loss of P3HB under irradiation at room temperature (blue) and irradiated in the molten state at 195 °C (red). At 0 kGy and 100%, data points overlap.

SEC showed a bimodal molar mass distribution with a number average molar mass M_n_ of 118,667 ± 5033 g/mol for the virgin P3HB (see Figure 10a and Figure 13a). Under irradiation at room temperature, the peak width decreased, and the main peaks shifted to smaller molecular masses. This is in agreement with the literature [11,16,45]. The longer chains are more likely to randomly scission under irradiation, and therefore, the distribution becomes more uniform while shifting to lower molar masses. The relative changes in the molar mass for the sample irradiated at room temperature (see Figure 11) are in agreement with the literature. For doses of up to 25 kGy, around 50%, and for doses of up to 50 kGy, around 80% of relative molar mass loss or higher are reported [15,45,46]. For a dose of 100 kGy, a relative mass loss of up to 90% is reported in this work. The decrease in polydispersity for the irradiation at room temperature is in accordance with the assumption of longer chains being more affected by the irradiation, and therefore, the molar mass distribution becoming more uniform (see Figure 10b). Since the radiation dose is defined as the energy absorbed by the irradiated materials per unit mass, the probability that a longer polymer chain absorbs more energy to generate polymer backbone radicals is higher in comparison to chains with lower molecular mass. Moreover, longer polymer chains are more limited in mobility by entanglement, through which the interaction to create a crosslink to a neighboring polymer chain is reduced, and therefore, chain scission reactions will be dominant.

Comparing the number average molar masses of virgin P3HB (PHB RT 0 kGy) M_n_ = 118 667 ± 5 033 g/mol with the P3HB, to which the heating cycle for the irradiation was applied (PHB 195 °C 0 kGy), a drop in the number average molar mass to M_n_ = 12,800 ± 346 g/mol due to thermal degradation can be seen (see Figure 9 and Figure 10a). When applying irradiation to the P3HB in the molten state, an initial drop in the number average molar mass to M_n_ = 9867 ± 351 g/mol for 50 kGy was determined. However, for a dose of 100 kGy, the number average molar mass rises back up to M_n_ = 11,000 ± 346 g/mol before dropping again for a dose of 150 to 9867 ± 404 g/mol and increasing to 10,267 ± 208 g/mol for 200 kGy. Since gel formation starts at 200 ± 55 kGy, GPC measurements might not account for higher molar mass fractions from crosslink formation. Due to this, the real number and mass average molar mass M_n_ and M_w_ for 200 kGy might be higher. By comparing the course of the molar mass distributions of the samples irradiated in a molten state in Figure 9b, a bulge for the sample irradiated with 100 kGy (PHB 195 °C 100 kGy) for higher molar masses is evident and bimodality starts to develop for 150 and 200 kGy. The rise in the mass average molar mass M_w_ (see Figure 10a) and the changes in the course of the graph are indications of branching occurring for the irradiation of P3HB in a molten state. The polydispersity of the samples irradiated in the melt constantly increases with the dose, while the average molar mass stays constant (see Figure 10a,b). For the relative molar mass in Figure 10, an increase to more than 350% for the mass average molar mass M_w_ can be seen. All of these are indications towards branching, since for random chain scission the polydispersity should constantly decrease as discussed above. The indications for branching reactions taking place in molten P3HB under irradiation align themselves with suspicions in other research, where branching occurred in poly(hydroxybutyrate–hydroxy valerate) (PHB/HV) depending on the ion source used in a solid state [47].

### 3.4. Rheology

**Figure 12:**
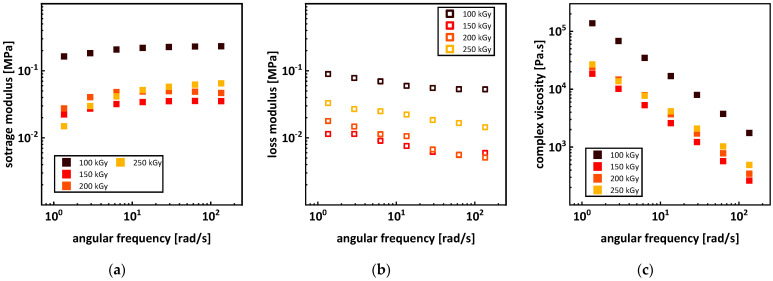
Storage (**a**) and loss (**b**) modulus, as well as complex viscosity (**c**) of P3HB irradiated in the molten state with different doses.

**Figure A2:**
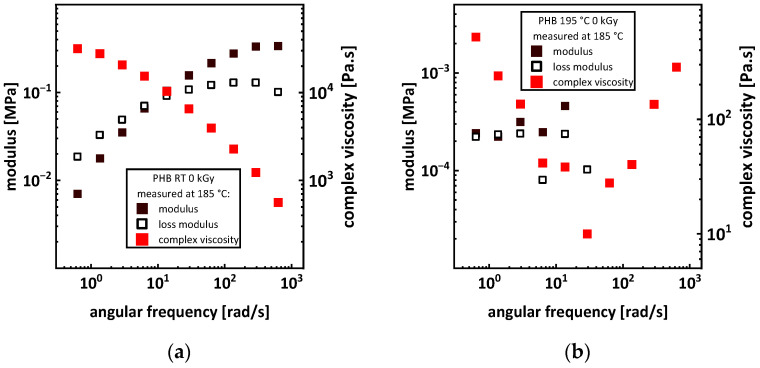
Rheological measurement of P3HB before the heating cycle (**a**) and after the heating cycle for irradiation processing (**b**).

Due to P3HB’s sensitivity towards thermal degradation, carrying out unrestricted rheological characterization was not possible. As shown in Figure 2, thermal degradation proceeds at different speeds regarding the initial molar mass [19]. This leads to different degradation rates for different applied doses since molar mass is strongly affected by irradiation. The differences in the melting point temperatures of up to 30 °C (see Figure 4) in combination with the differences in molar masses (see Figure 10) complicate the comparability of the rheological measurements. For higher melting samples, temperatures could not be lowered due to crystals forming in the melt, and for lower melting samples, temperatures could not be raised because thermal degradation would increase severely, and viscosity would lower to such an extent that no conclusive data could be obtained (see Appendix A and Figure A2). The limited variety in temperatures for measurements also did not allow a determination of master curves to directly compare the rheological properties in an overview. However, through frequency sweeps from 135 to 1 rad/s at 165 °C, changes in the storage and loss modulus as well as the complex viscosity for doses between 100 and 250 kGy applied in the molten state were determined (see Figure 12). For the samples irradiated at room temperature, the viscosity at 165 °C was too low for measurements to reveal valid data, so no comparison was possible. The low bulk density of the P3HB powder also led to problems. The low bulk density limited the amount of a sample’s mass to 2 g per mold; therefore, only very thin discs could be obtained. The thin disc limited the gap for rheological measurements severely. Adding more sample mass after melting led to air inclusions in the discs, therefore making them unsuitable for rheological measurements. Previous injection molding or pressing of the powder for irradiation treatment would add additional thermal strain, leading to even more thermal degradation, even to the point where effects of the irradiation in the molten state would no longer have any impact.

**Figure 13:**
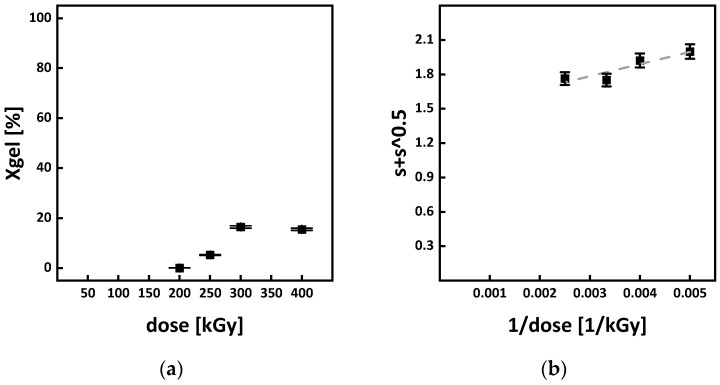
(**a**) shows the gel content of and (**b**) the Charlesby–Pinner plot for P3HB irradiated in the molten state at 195 °C. For linear function of the plot refer to Equation (6).

For an increasing dose of 100 to 150 kGy, the storage modulus, the loss modulus, and the complex viscosity decreased (see Figure 12). For doses of 150 to 250 kGy, the storage modulus increased for higher frequencies and decreased for lower frequencies. After the drop from 100 to 150 kGy, the loss modulus and the complex viscosity increased for 150 to 250 kGy. Branching leads to an increase in complex viscosity, so the increase from 150 to 250 kGy can be interpreted as an increase in branching (see Figure 12c). This is consistent with the data obtained from SEC (see Figure 9b), where a shift to higher mass average molar masses M_w_ can be seen for doses from 150 to 200 kGy. At around 200 kGy, gel formation starts and gel content reached 5% at 250 kGy (see Figure 13a). For crosslinked polymers, the complex viscosity should increase for lower frequencies and the slope of the curve should increase. Likewise, the storage modulus should increase for lower frequencies and the loss modulus should decrease overall when network formation sets in. This seems not to be the case as seen in Figure 12. The network response is also dependent on the phases and molar masses surrounding the network, influencing the changes in the course of the moduli and the complex viscosity. A second factor is the low average molar mass M_n_ below 1200 g/mol (see Figure 10a). The low molar mass fractions in the P3HB diffuse to the sample–plate interface and hinder energy transfer into the material. These two factors explain the disparity between the rheological results and data from SEC and the gel content. Furthermore, a network content of 5% might not be high enough for this system to create a proper response during rheological measurements.

### 3.5. Gel Formation and G Values

When irradiated at room temperature, no gel formation and therefore no crosslinking in P3HB could be detected. For the irradiation in the molten state at 195 °C and doses above 200 kGy, gel formation started to occur (see Figure 13a). For doses above 300 kGy, a maximum gel content of around 16% was reached. The Charlesby–Pinner plot is depicted in Figure 13b and the solution is determined to be:

(9) $$s+\sqrt{s}=\left(1.47\;\pm 0.12\right)+\left(105.98\pm 32.09\right)\;\;\frac{1}{D}$$

With $s+\sqrt{s}=2$ and according to Equation (9), the gel dose D_gel_ is 200 ± 55 kGy for irradiation in a molten state. The large error obtained displays the limited viability of the Charlesby–Pinner equation due to the polydispersity being higher than two (see Figure 10). Since no gel dose value for P3HB can be found in the literature, comparing the value obtained here to those of similar polyesters is necessary to put it into perspective. For polylactide acid (PLA) irradiated in a molten state at 170 °C, a gel dose of around 150 kGy can be found [15,24]. For the irradiation of 100% amorphous PLA above its glass transition temperature, similar gel doses were reported [23]. Assuming similar chain mobility due to the same side group in P3HB and PLA and taking into consideration that PLA also mainly undergoes chain scission when irradiated at room temperature [48], it is argued that the gel dose obtained for P3HB is reasonable.

Table 1 displays the molar masses of the virgin P3HB (PHB 0 kGy RT) and of the non-irradiated P3HB after the heating cycle for irradiation processing (PHB 0 kGy 195 °C). It is argued that by applying the molar mass of the virgin P3HB to the Charlesby–Pinner equation to determine the G values, thermal degradation is not accounted for. When performing the calculations with the molar mass of the heated P3HB, scission, due to thermal degradation, also contributes, and therefore, G(S) is overestimated. Depending on what molar mass M_n_ the determination of the G values is based on, the molecular mass of the virgin P3HB or that after applying the heating cycle, different values for G(S) and G(X) were obtained (see Table 1). In the literature, the values for G(S) in P3HB under irradiation in a solid state range from 5.4 to 15.7 scissions/100 eV [15,16,36]. These values were obtained through viscosity average molar mass determination in an Ostwald-type capillary viscometer by dissolving the P3HB in chloroform. Compared to the literature, the G(S) value of 10.45 scissions/100 eV, obtained from the molar mass of 12,800 ± 346 g/mol after applying the heating cycle, appears more realistic. However, a true determination of a sole irradiation-based G(S) is not possible since the 10.45 scissions/100 eV include thermal degradation. It is argued that the two G(S) values shown in Table 1 are the limits to which the real G(S) value is located when irradiation is carried out in a molten state. A similar argument applies to the G(X) values. For increasing molar masses, the G(X) value decreases; however, in the case of higher chain mobility, due to decreasing molar masses, the G(X) value increases. Due to the applied heating for melting during the irradiation in this study, the molar mass decreases and therefore the G(X) value increases. However, since crosslinking reactions dominate in the range from 200 to 400 kGy, a shift to lower values for G(S) and to higher values for G(X) seems plausible. This would shift the ratio of G(S) and G(X) more towards crosslinking and therefore explain the gel formation. Form Table 1, a range for the ratio of G(X)/G(S) from 0.04 to 3.14 can be calculated.

The gel dose of 200 ± 55 kGy reported in this work is higher than a potential gel dose found by Bergmann et al. [11]. Bergmann et al. [11] reported indications for crosslink formation in the amorphous phase of P3HB at a dose of 33 kGy. This discrepancy comes down to thermal degradation. Comparing the molar masses in Table 1 shows a drop in molar mass of around 90% when applying the heating cycle for irradiation. Therefore, a higher dose and more energy are needed for the crosslink reactions to overcome the thermal degradation. If thermal degradation is less severe, a lower gel dose for the irradiation in the molten state will be reported.

**Figure 14:**
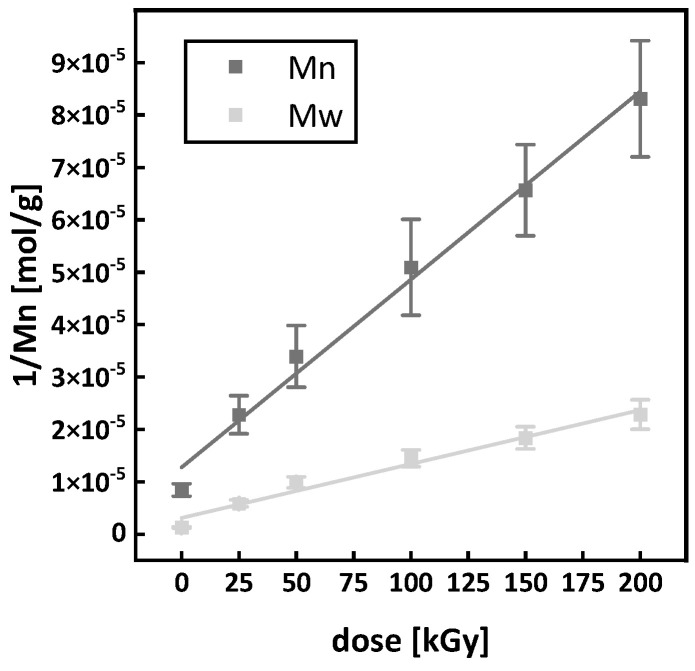
Solution of Equations (7) and (8) for P3HB irradiated at room temperature.

For the irradiation at room temperature, the solutions to Equations (7) and (8) are shown in Figure 14. The graphical solutions for the equations are

(10) $$y=\left(\left(1.27\times 10^{-5})\;\pm (0.20\times 10^{-5}\right)\right)+\left(\left(3.59\times 10^{-7})\pm (0.18\times 10^{-7}\right)\right)\;\;D$$

for Equation (7) with an R-squared of 0.99 and

(11) $$y=\left(\left(3.12\times 10^{-6})\;\pm (0.91\times 10^{-6}\right)\right)+\left(\left(1.03\times 10^{-7})\pm (0.08\times 10^{-7}\right)\right)\;\;D$$

for Equation (8) with an R-squared of 0.98. By inserting the values of the slope and the y-intercept in Equations (7) and (8), a G(S) value of 4.1 ± 0.5 scissions/100 eV and a G(X) value of 0.6 ± 0.2 crosslinks/100 eV for the irradiation at room temperature are obtained. Comparing the G(S) for the irradiation at room temperature to the literature, the value presented in this work (G(S) 4.1 ± 0.5) is below the values of 5.4 to 15.7 scissions/100 eV given [13,32,33] but lies in the range given in Table 1. Furthermore, the calculated G(S) value for the irradiation at room temperature (4.1 ± 0.5) is found in the lower part of the range (1.13 to 10.45) as proposed. This calculation is further proof that the assumptions made for the Charlesby–Pinner plot to calculate the range for the G(S) value are valid. For a better overview, G(S) values from this work and values given in the literature are also displayed in Table 2. The calculated G(X) value for the irradiation at room temperature of 0.6 ± 0.2 crosslinks/100 eV is also inside the range for G(X) 0.38 ± 0.12 to 3.55 ± 1.08 shown in Table 1. It was proposed that the true G(X) value would be closer to the upper limit of the range. At first sight, this seems not to be the case, but since the value of 0.6 ± 0.2 crosslinks/100 eV was obtained from irradiation in a solid state, it does not account for extended chain mobility in the melt. The higher chain mobility in the melt enables radicals to find partners for interactions faster and more easily. Taking this into account, 0.6 ± 0.2 crosslinks/100 eV is also just a lower limit for the true G(X) value and, furthermore, supports the arguments made for the considerations of the Charlesby–Pinner plot, namely using the molar masses before and after the heating cycle for calculation.

## 4. Conclusions

In this work, we were able to induce branching and crosslinking in P3HB through e-beam irradiation in a molten state and are closing a gap in science and the literature concerning the irradiation of P3HB in a non-crystalline state. Through DSC, TGA, SEC, and rheological measurements, we conclusively showed that branching reactions begin to occur around 100 kGy. The gel dose was determined to be 200 ± 55 kGy and a maximum gel content of 16% was reached at around 300 kGy. The courses of branching reactions as well as the course of network formation depending on the irradiation dose are described. For the first time, thermal degradation during irradiation was taken into consideration for G value determination and ranges for the G values were calculated, namely 1.13 to 10.45 scissions/100 eV for G(S) and 0.38 to 3.55 crosslinks/100 eV for G(X), and a comparison of G values from this work to the literature is shown. P3HB’s sensitivity to thermal degradation was the main limiting factor in this work concerning irradiation processing at elevated temperatures as well as rheological characterization. In particular, the molar mass decrease due to heating generated problems during rheological measurements, making the obtained data difficult to compare. We, however, were able to correlate the rheological measurements with insights from DSC, TGA, and SEC and therefore evince conclusively the molecular and structural changes in P3HB when increasing irradiation in a molten state.

## Figures and Tables

**Table 1:** G values of P3HB for irradiation in the molten state at 195 °C. Calculated from the Charlesby–Pinner equation. Two values for M_n_ were taken for the calculation to account for thermal degradation. Adjusted G values are proposed to be in the range given in this table.

M_n_ (g/mol)	G(S) (Scissions/100 eV)	G(X) (Crosslinks/100 eV)
118,667 ± 5033	1.13 ± 0.18	0.38 ± 0.12
12,800 ± 346	10.45 ± 1.64	3.55 ± 1.08

**Table 2:** Comparison of G values from this work and those obtained from literature. G(S) values from [15,16,36] were obtained through the changes in the viscosity average molecular weight. All films in this table were produced by solution casting.

Source	This Work		**[16]**		**[15]**		**[36]**	
Material	PHB		PHB	PHBV	PHB		PHB	
	powder		film	film	powder	film	powder	film
irradiation temperature	room temperature	195 °C	room temperature		room temperature		room temperature	
G(S) (scissions/100 eV)	4.1	1.13 to 10.45	15.7	12.9	15.7	5.4	5.9	6.0
G(X) (crosslinks/100 eV)	0.6	0.38 to 3.55	-	-	-	-	-	-

## Data Availability

The data presented in this study are available on request.

## Appendix A

### Appendix A.1. Thermal Analysis

The DSC curves of the first heating cycle of P3HB irradiated at room temperature and in the melt are shown in Figure A1. Important datapoints from the first and the second heating cycles are displayed in Table A1. Datapoints from TG measurements are shown in Table A2.

**Table A1:** Datapoints obtained from DSC measurements from both cycles. Some of the DSC curves in Figure A1 and Figure 4 show two peaks while melting. Main and side peaks are listed separately.

heating rate	first cycle																																						
10 K/min	heating															cooling																							
	Tm															Tg												Tc											
	peak area			main peak			minor peak			onset			end			onset			middle point			turning point			end			peak area			peak			onset			end		
sample	[J/g]			[°C]			[°C]			[°C]			[°C]			[°C]			[°C]			[°C]			[°C]			[J/g]			[°C]			[°C]			[°C]		
PHB RT 0 kGy	103.4	±	5.2	177	±	9		-		163	±	8	184	±	9		-			-			-			-		−78.7	±	3.9	82	±	4	76	±	4	89	±	4
PHB RT 25 kGy	97.1	±	4.9	173	±	9		-		161	±	8	178	±	9		-			-			-			-		−67.4	±	3.4	75	±	4	#	±	3	99	±	5
PHB RT 50 kGy	98.9	±	4.9	171	±	9		-		162	±	8	176	±	9		-			-			-			-		−74.0	±	3.7	76	±	4	59	±	3	89	±	4
PHB RT 100 kGy	98.4	±	4.9	166	±	8		-		158	±	8	170	±	8		-			-			-			-		−67.7	±	3.4	66	±	3	51	±	3	88	±	4
PHB RT 150 kGy	98.1	±	4.9	163	±	8		-		156	±	8	167	±	8		-			-			-			-		−69.6	±	3.5	64	±	3	#	±	2	91	±	5
PHB RT 200 kGy	96.0	±	4.8	161	±	8		-		154	±	8	165	±	8		-			-			-			-		−64.4	±	3.2	60	±	3	#	±	2	83	±	4
PHB RT 250 kGy	93.3	±	4.7	159	±	8		-		153	±	8	162	±	8		-			-			-			-		−61.4	±	3.1	57	±	3	#	±	2	74	±	4
PHB RT 300 kGy	89.8	±	4.5	157	±	8		-		152	±	8	161	±	8		-			-			-			-		−52.6	±	2.6	56	±	3	#	±	2	71	±	4
PHB 195 °C 0 kGy	102.3	±	5.1	171	±	9	162	±	8	167	±	8	173	±	9		-			-			-			-		−77.6	±	3.9	77	±	4	#	±	3	84	±	4
PHB 195 °C 25 kGy	96.1	±	4.8	168	±	8	157	±	8	162	±	8	171	±	9		-			-			-			-		−76.2	±	3.8	76	±	4	#	±	3	84	±	4
PHB 195 °C 50 kGy	97.1	±	4.9	165	±	8	153	±	8	160	±	8	168	±	8		-			-			-			-		−76.4	±	3.8	70	±	3	61	±	3	79	±	4
PHB 195 °C 100 kGy	86.3	±	4.3	161	±	8	147	±	7	157	±	8	164	±	8		-			-			-			-		−72.8	±	3.6	67	±	3	56	±	3	80	±	4
PHB 195 °C 150 kGy	73.5	±	3.7	156	±	8	141	±	7	152	±	8	159	±	8		-			-			-			-		−58.1	±	2.9	59	±	3	#	±	2	72	±	4
PHB 195 °C 200 kGy	71.2	±	3.6	154	±	8	137	±	7	150	±	8	156	±	8	−7	±	1	−2	±	1	−2	±	1	2	±	1	−31.1	±	1.6	54	±	3	37	±	2	69	±	3
PHB 195 °C 250 kGy	61.0	±	3.0	149	±	7	130	±	7	143	±	7	152	±	8	−8	±	1	−2	±	1	−2	±	1	2	±	1	−12.0	±	1.0	51	±	3	35	±	2	66	±	3
PHB 195 °C 300 kGy	55.8	±	2.8	146	±	7	127	±	6	141	±	7	150	±	7	−9	±	1	−3	±	1	−3	±	1	1	±	1	−2.7	±	1.0	49	±	2	#	±	2	62	±	3

second cycle																																																														
heating																																							cooling																							
Tg												Tcc												Tm															Tg												Tc											
onset			middle point			turning point			end			peak area			main peak			onset			end			peak area			main peak			minor peak			onset			end			onset			middle point			turning point			end			peak area			peak			onset			end		
[°C]			[°C]			[°C]			[°C]			[J/g]			[°C]			[°C]			[°C]			[J/g]			[°C]			[°C]			[°C]			[°C]			[°C]			[°C]			[°C]			[°C]			[J/g]			[°C]			[°C]			[°C]		
	-			-			-			-			-			-			-			-		104.1	±	5.2	173	±	9		-		165	±	8	181	±	9		-			-			-			-		−82.9	±	4.1	83	±	4	77	±	4	89	±	4
	-			-			-			-			-			-			-			-		95.8	±	4.8	172	±	9	164	±	8	166	±	8	178	±	9		-			-			-			-		−81.2	±	4.1	75	±	4	64	±	3	97	±	5
	-			-			-			-			-			-			-			-		93.6	±	4.7	170	±	9	160	±	8	163	±	8	176	±	9		-			-			-			-		−78.4	±	3.9	77	±	4	61	±	3	94	±	5
	-			-			-			-			-			-			-			-		87.6	±	4.4	165	±	8	153	±	8	159	±	8	168	±	8		-			-			-			-		−74.6	±	3.7	76	±	4	56	±	3	98	±	5
	-			-			-			-			-			-			-			-		82.7	±	4.1	162	±	8	149	±	7	155	±	8	165	±	8		-			-			-			-		−73.3	±	3.7	76	±	4	54	±	3	97	±	5
0	±	1	2	±	1	3	±	1	4	±	1	−2.6	±	1.0	48	±	2	39	±	2	55	±	3	78.5	±	3.9	159	±	8	145	±	7	152	±	8	162	±	8		-			-			-			-		−65.7	±	3.3	63	±	3	48	±	2	90	±	5
1	±	1	3	±	1	2	±	1	4	±	1	−4.8	±	1.0	48	±	2	40	±	2	55	±	3	77.1	±	3.9	156	±	8	141	±	7	150	±	7	160	±	8		-			-			-			-		−70.3	±	3.5	75	±	4	47	±	2	98	±	5
0	±	1	2	±	1	2	±	1	3	±	1	−7.1	±	1.0	49	±	2	39	±	2	56	±	3	69.9	±	3.5	154	±	8	143	±	7	148	±	7	158	±	8		-			-			-			-		−62.4	±	3.1	66	±	3	44	±	2	94	±	5
	-			-			-			-			-			-			-			-		100.7	±	5.0	167	±	8	156	±	8	150	±	8	171	±	9		-			-			-			-		−77.6	±	3.9	75	±	4	68	±	3	83	±	4
	-			-			-			-			-			-			-			-		96.0	±	4.8	165	±	8	152	±	8	153	±	8	168	±	8		-			-			-			-		−76.5	±	3.8	74	±	4	66	±	3	88	±	4
	-			-			-			-			-			-			-			-		98.0	±	4.9	161	±	8	148	±	7	155	±	8	165	±	8		-			-			-			-		−78.3	±	3.9	71	±	4	60	±	3	97	±	5
	-			-			-			-			-			-			-			-		88.9	±	4.4	157	±	8	143	±	7	150	±	8	161	±	8		-			-			-			-		−72.2	±	3.6	657	±	33	55	±	3	78	±	4
	-			-			-			-		−2.9	±	1.0	49	±	2	40	±	2	55	±	3	77.4	±	3.9	152	±	8	136	±	7	144	±	7	156	±	8		-			-			-			-		−58.0	±	2.9	58	±	3	43	±	2	71	±	4
0	±	1	1	±		2	±	1	3	±	1	−24.8	±	1.2	53	±	3	43	±	2	59	±	3	77.0	±	3.8	150	±	7	132	±	7	141	±	7	154	±	8	−6.7	±	1	−2	±	1	−2.7	±	1	1.7	±	1	−35.7	±	1.8	54	±	3	37	±	2	71	±	4
0	±	1	1	±		1	±	1	3	±	1	−39.9	±	2.0	56	±	3	47	±	2	63	±	3	66.7	±	3.3	146	±	7	126	±	6	136	±	7	151	±	8	−7.4	±	1	−2	±	1	−2.4	±	1	1.5	±	1	−11.4	±	0.6	50	±	3	34	±	2	66	±	3
−1	±	1	1	±		1	±	1	2	±	1	−49.8	±	2.5	59	±	3	49	±	2	65	±	3	63.3	±	3.2	140	±	7	123	±	6	133	±	7	148	±	7	−8.3	±	1	−3	±	1	−4.4	±	1	0.5	±	1	−2.3	±	0.1	49	±	2	34	±	2	65	±	3

**Table A2:** Data points from TG measurements.

	**Temperature [°C]**																	
**Dose**	**1% Mass Loss**			**5% Mass Loss**			**Turning Point**			**1% Mass Loss**			**5% Mass Loss**			**Turning Point**		
[kGy]	irradiation at 195 °C									irradiation at room temperature								
0	254	±	6	269	±	7	292	±	7	259	±	6	274	±	7	294	±	7
100	240	±	6	267	±	7	287	±	7	253	±	6	270	±	7	292	±	7
200	242	±	6	266	±	7	291	±	7	241	±	6	266	±	7	289	±	7
300	227	±	6	257	±	6	289	±	7	241	±	6	264	±	7	287	±	7

### Appendix A.2. Rheology

Measurements for the virgin P3HB and the P3HB with one heating cycle for processing applied are displayed in Figure A2. Both measurements were taken at the same temperature, namely 185 °C, and the same procedure for the measurements was applied. It is evident that due to the molar mass decrease in P3HB during the heating cycle, the melting points of the two samples are so far apart that a direct comparison is not possible. It is also not possible to measure master curves since the temperature range returning sensible data for measuring is too narrow.
